# Treatment of inferior pole fracture of the patella with tension-free external immobilization

**DOI:** 10.1186/s12893-022-01790-x

**Published:** 2022-09-12

**Authors:** Shaoquan Pu, Yanling Chen, Jinlong Liang, Yongqing Xu, Yonghui Zhao

**Affiliations:** 1 Department of Orthopedics, 920 Hospital of the Joint Logistic Support Force, 650032 Kunming, China; 2 Department of Orthopedics, The First People’s Hospital of Yunnan Province, The Affiliated Hospital of Kunming University of Science and Technology, 650032 Kunming, China

**Keywords:** External immobilization, Inferior pole fracture of patella, Knee

## Abstract

**Background:**

Inferior pole fracture of the patella (IPFP) has small and comminuted fracture blocks that are hard to immobilize, and early mobilization may lead to loss of fracture reduction and immobilization failure. Therefore, a difficulty of treatment is to achieve rigid immobilization with early functional exercise. Here, a new treatment method of tension-free external immobilization is put forward.

**Methods:**

The clinical data of 11 IPFP patients treated with tension-free external immobilization from May 2016 to June 2019 were retrospectively analyzed. There were six males and five females aged 39.0 ± 12.8 years (range 18–53 years). IPFP was caused by traffic accidents in five cases and falls in six cases. All cases had unilateral closed injuries, including four in the left knee and seven in the right knee. The preoperative range of motion of the knee was 22.0 ± 7.5° (10–30°). The time from injury to operation was 4.5 ± 1.3 d (3–7 d). The operation-related indices were recorded, and the function of the affected knee was assessed by the Böstman score.

**Results:**

All operations were successful. The operation time was 56.4 ± 8.4 mi (45–70 min), the intraoperative blood loss was 54.1 ± 14.6 mL (40–80 mL), and the length of hospital stay was 7.5 ± 1.9 d (5–11 d). The mean follow-up time was 20.4 ± 7.6 months (12–36 months), the duration of fracture healing was 8.9 ± 1.5 weeks (7–12 weeks), and the removal time of the external immobilization device was 10.4 ± 0.9 weeks (9–12 weeks). At the last follow-up, the range of motion had no significant difference between the affected knee (129.7 ± 3.3°, range 125–135°) and the unaffected knee (130.8 ± 3.8°, range 126–137°) (*t* = 0.718, *p* < 0.05). The Böstman score of the knee was 29.2 ± 1.0 points (27–30 points), including 10 excellent cases (90.9%) and one good case (9.1%).

**Conclusion:**

Tension-free external immobilization is a feasible treatment for IPFP. It can help with early functional exercise and achieve a satisfactory clinical effect.

## Introduction

As a common intra-articular fracture, patellar fracture accounts for about 1% of systemic fractures [1]. Inferior pole fracture of the patella (IPFP) is a special type of patellar fracture occurring in the distal 1/4 of the patella, i.e., the point of attachment of the patellar tendon, made up mainly of cancellous bone with no articular surface coverage and not involved in the composition of the patellofemoral joint. As an extra-articular fracture [2], IPFP makes up 9.3–22.4% of patellar fractures [3].

IPFP has small and comminuted fracture blocks that are hard to immobilize, and it is also prone to displacement due to patellar tendon traction, so conservative treatment usually has unsatisfactory effects, necessitating surgical intervention. Currently, IPFP is primarily treated with two surgical methods. The first method is inferior patellar pole resection and patellar ligament repair and reconstruction, but this shortens the patellar ligament, leads to patellar lowering, and increases the pressure on the patellofemoral joint surface, resulting in complications such as limited knee flexion and anterior patellar pain [4]. The second method is reduction, and immobilization with steel wires, steel plates and sutures, aiming to preserve the anatomical integrity of the patella [5–9], but it has limited stability in the immobilization of smaller IPFPs, and early mobilization may lead to loss of fracture reduction and immobilization failure [9, 10, 11, 12]. Therefore, how to restore the knee function through early rehabilitation exercise at the same time as effective immobilization remains a clinical problem demanding a prompt solution.

This study describes the characteristics of the new tension-free external immobilization device and retrospectively assesses its clinical effect in the treatment of IPFP.

## Materials and methods

### General information

Inclusion criteria: (1) unilateral IPFP diagnosed by imaging, (2) age ≥ 18 years, (3) normal bone mineral density, and 4) ≥ 12 months of follow-up. Exclusion criteria: (1) pathological fracture, (2) fracture of the femur, tibia, or fibula on the affected side, (3) popliteal blood vessel or nerve injury, (4) other acute or chronic diseases affecting knee function, and (5) multiple injuries or intolerance to surgery due to underlying diseases.

Six males and five females aged 39.0 ± 12.8 years (range 18–53 years) were included in the study. IPFP was caused by traffic accidents in five cases and falls in six cases. All cases had unilateral closed injuries, including four injuries to the left knee and seven to the right knee. The preoperative range of motion of the knee was 22.0 ± 7.5° (10–30°). The time from injury to operation was 4.5 ± 1.3 d (3–7 d).

The same surgical team performed all the surgeries in this study. This study complied with the Declaration of Helsinki and was approved by the Ethics Committee of the 920 Hospital of Chinese People’s Liberation Army Joint Logistics Support Force. We obtained informed consent from all subjects and/or their legal guardian(s).

### Surgical techniques

**Fig. 1:**
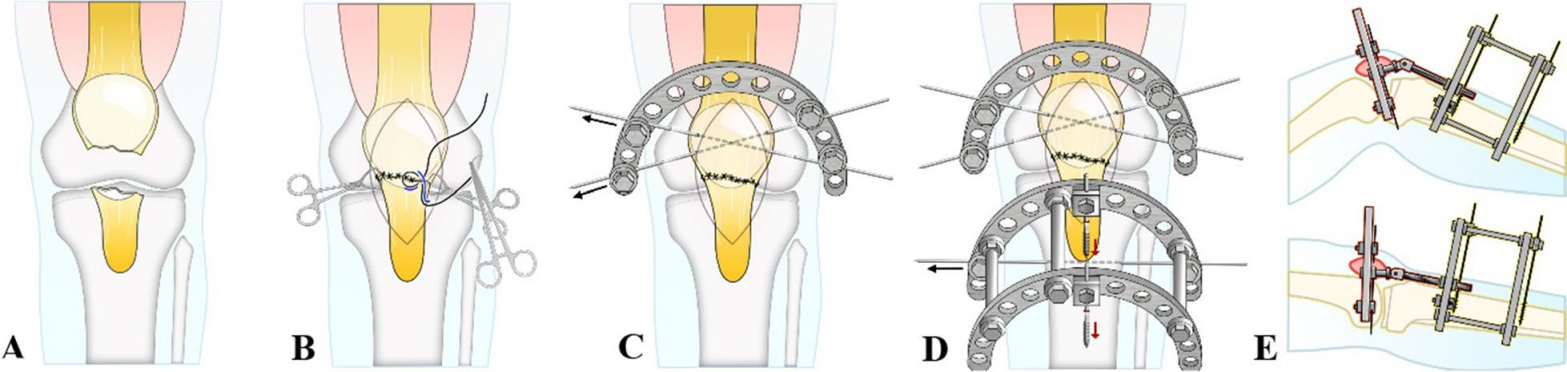
Procedures of tension-free external immobilization. **A** An anterior median incision of the patella was made to expose the fracture end. **B** The fracture end was sutured in a knee-extension position. **C** Kirschner wires were cross-inserted in the vertical direction of the waist segment of the patella and fixed with the ^1/2^ ring. **D** The two ^2/3^ rings were bridged and fixed with Kirschner wires and threaded needles on the plane of tibial tubercle and two distal planes. **E** The devices at the patellar end and the tibial end were connected and fixed using the joint hinge, and the joint motion and tension-free state of the fracture end were maintained

The tension-free external immobilization device was mainly composed of Kirschner wire, threaded needle, external immobilization ring (including and 1/2 ring and a 2/3 ring), threaded rod, plain bolt, needle-passing bolt, threaded needle pad, joint hinge, and vertical linker (Fig. 1).

After general anesthesia in the supine position, routine disinfection, and draping, a sterile tourniquet was put on the root of the thigh on the affected side and inflated. An anterior median longitudinal incision of the patella was made to expose the IPFP end (Fig. 1A). The fracture ends were handled under direct vision. If the avulsion fracture blocks were large, fracture immobilization and reduction were performed using olive-tipped needles or Kirschner wires with a blocking head. One-gauge absorbable sutures were used for suture immobilization (Fig. 1B). Two 1.5-mm Kirschner wires were cross-inserted percutaneously in the vertical direction of the waist segment of the patella, and the Kirschner wires and the 1/2 ring were fixed with needle-passing bolts (Fig. 1C). One 2.0-mm Kirschner wire was inserted below the tibial tubercle horizontally through the tibia, one 4.0-mm threaded needle was inserted vertically in front of the tibia on this plane, and the Kirschner wire, threaded needle, and 2/3 ring were connected and fixed with needle-passing bolts and threaded needle pads. One 4.0-mm threaded needle was inserted vertically in front of the tibia about 12 cm away from the distal end of the 2/3 ring, and the two 2/3 rings were bridged and fixed with threaded needles, needle-passing bolts, and threaded needle pads (Fig. 1D). Then the devices at both ends of the fracture were connected using the joint hinge, and the joint hinge was adjusted to allow for the good knee motion and no tension of the suture at the fracture end (Fig. 1E). Finally, the tourniquet was loosened, the incision was washed, the bleeding was stopped, and the surgical incision was closed.

### Postoperative care and follow-up

Within 24 h after surgery, we gave antibiotics to prevent infection and nonsteroidal anti-inflammatory drugs to control pain. The knee could be passively moved on the bed after surgery. The patients were encouraged to actively move the knee 1 d after surgery, they strengthened the knee function and walked on crutches with partial weight at 2 d, and they walked with no crutches at full weight at 1 week.

The anteroposterior and lateral X-rays of the knee on the affected side were reviewed within 3 d after surgery first, once a month until fracture healing and removal of external immobilization devices, and then once every 6 months. The operation duration, intraoperative blood loss, length of hospital stay, and surgical complications were recorded, and the fracture healing time, removal time of external immobilization device, and postoperative complications were recorded during the follow-up. Before surgery, 1 month after surgery, and at the last follow-up, the range of motion of the knee was measured. At the last follow-up, the range of motion was compared between the affected knee and unaffected knee, and the function of the affected knee was assessed by the Böstman score.

### Statistical analysis

SPSS 24.0 software (SPSS, USA) was used for analysis. Measurement data are expressed as ($\overline {\text{x}}$ ± s). The range of motion of the knee was compared at different time points through the paired *t*-test. The test level was set as α = 0.05.

## Results

All operations were successful. The operation time was 56.4 ± 8.4 min (45–70 min), the intraoperative blood loss was (54.1 ± 14.6) mL (40–80 mL), and the length of hospital stay was 7.5 ± 1.9 d (5–11 d). Redness and swelling occurred in the needle tract in three cases during the postoperative frame-carrying period, and it was handled by dressing changes and wet dressing with alcohol. Good needle tract healing was achieved within 1 week after removal of the external immobilization frame. The patients were followed up for 20.4 ± 7.6 months (12–36 months) and had fracture healing after 8.9 ± 1.5 weeks (7–12 weeks). The external immobilization device was removed at 10.4 ± 0.9 weeks (9–12 weeks).

**Fig. 2:**
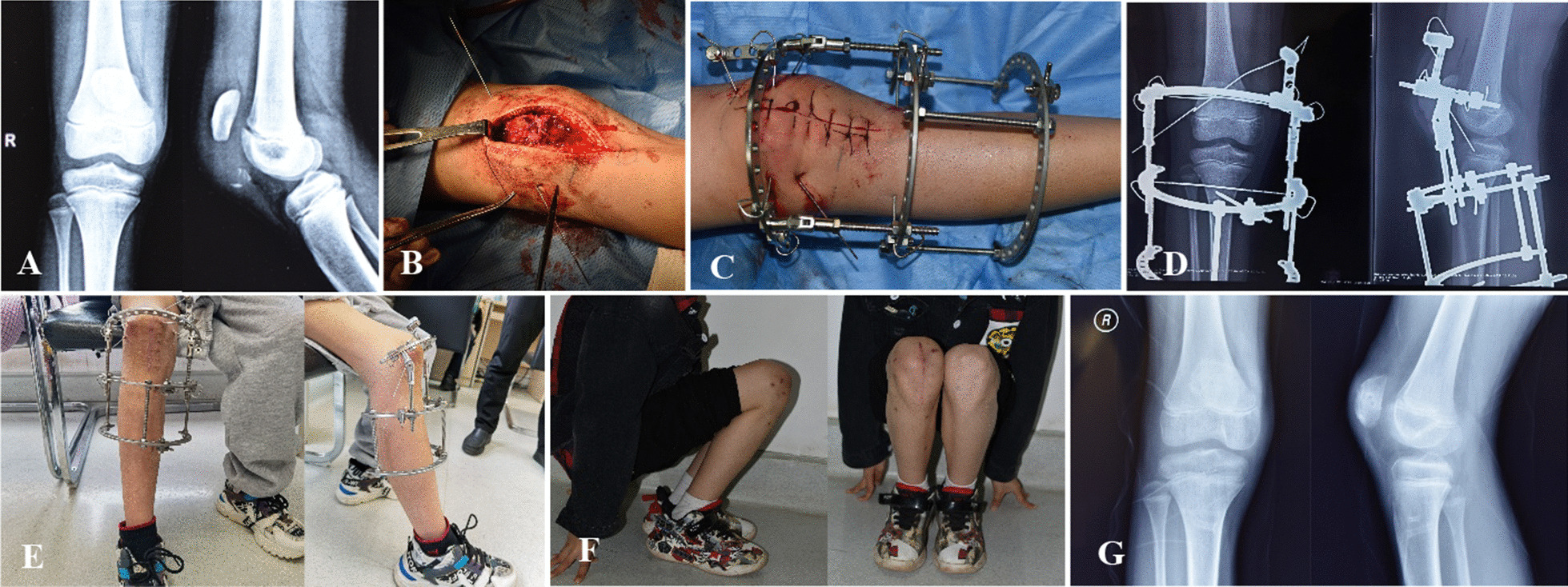
A 18-year-old male patient with right IPFP due to a fall. **A** Anteroposterior and lateral X-rays of the right knee before surgery. **B** After avulsion fracture reduction, the fracture block was immobilized using Kirschner wires with a blocking head, and 1-gauge absorbable sutures were used for suture immobilization. **C** Postoperative tension-free external immobilization device. **D** Anteroposterior and lateral X-rays of the right knee after surgery. **E** Postoperative frame-carrying conditions. **F** The knee function was good after removal of the external immobilization frame. **G** Anteroposterior and lateral X-rays of the right knee after removal of the external immobilization frame

The range of motion of the knee was greatly improved at 1 month after surgery [88.6 ± 11.4° (70–105°)] compared with before surgery [22.0 ± 7.5° (10–30°)] (*t* = 16.187, *p* < 0.05). At the last follow-up, the range of motion of the knee [129.7 ± 3.3° (125–135°)] was further improved compared with that at 1 month after surgery (*t* = 11.474, *p* < 0.05) and had no significant difference from that on the unaffected side [130.8 ± 3.8° (126–137°)] (*t* = 0.718, *p* < 0.05). At the last follow-up, the Böstman score of the knee was 29.2 ± 1.0 points (27–30 points), including 10 excellent cases (90.9%) and one good case (9.1%). During the follow-up period, no complications, such as loss of fracture reduction, internal immobilization failure, or joint stiffness, occurred (Fig. 2).

## Discussion

IPFP has small and severely comminuted fracture blocks that are hard to immobilize. No consensus has been reached on the treatment of IPFP, and there are pros and cons for each treatment method reported in the literature [13]. Most treatments entail resection or reduction of fracture blocks. Most scholars believe that reduction of fracture blocks can restore the normal anatomical structure of the patella to the greatest extent, while resection of fracture blocks will result in patellar defect, patellofemoral joint dislocation, high tension of the patellar tendon, and difficulty with tendon-bone healing [4]. Fracture blocks are preserved and reduced mostly by internal immobilization using steel plates, steel wires, and sutures. Patellar concentrators are a commonly used immobilization device in the clinic that can concentrically gather the displaced patellar fracture blocks [6], but they are less effective in the immobilization of smaller IPFPs. Basket plates can effectively gather the fracture blocks and restore the knee extension function [5, 14], but they cause great damage to the structure and function of the patellar ligament, such as shortening of the patellar ligament, destruction of blood supply of the patellar ligament, and internal immobilization irritation during knee flexion [14, 15, 16]. Due to dispersed cohesion, traditional steel wire or suture cerclage fails to create synergy in immobilization and causes unstable immobilization, and fracture block separation and rotational displacement occur easily during knee flexion and extension [17]. Postoperative auxiliary plaster immobilization is prone to cause knee stiffness and knee functional limitation. Silk thread or suture anchors can be used to suture the patellar fracture blocks, but they fail to provide enough strength for early functional exercise [18]. Tension bands with Kirschner wires or cannulated screws can effectively immobilize the fracture blocks and antagonize the tension of anterior patellar ligament, facilitating postoperative early functional exercise and yielding good prognoses [19]. However, they behave poorly in the immobilization of IPFP and are prone to loosening after surgery, leading to internal immobilization failure [19, 20]. At present, rigid immobilization with early functional exercise following IPFP reduction remains a difficulty [21, 22].

In view of the difficulty treating IPFP and the limitations of the above surgical methods, we put forward a new treatment method tension-free external immobilization. The external immobilization device includes a patellar immobilization part and a tibial immobilization part, as well as a joint hinge connecting the two parts. This method can achieve the stable immobilization in 3D space, and the sutured fracture end can be in a tension-free state (by adjusting the joint hinge during surgery) while ensuring the good range of motion of the knee. In this study, good fracture healing was achieved in all 11 patients after tension-free external immobilization. Redness and swelling occurred in the needle tract in three cases during the postoperative frame-carrying period, and it was healed by symptomatic treatment within 1 week after removal of the external immobilization frame. No other surgical complications occurred. At the last follow-up, the Böstman score of the knee was 29.2 ± 1.0 points, including 10 excellent cases and one good case, suggesting satisfactory clinical efficacy.

As can be seen from the technical characteristics, the advantages of the tension-free external immobilization are as follows: (1) Tension-free immobilization. With the upper end fixed at the waist segment of the patella and the lower end fixed at the upper segment of the tibia, the fracture end can be reduced and immobilized in a tension-free state. By adjusting the joint hinge, the knee motion of the fracture end can be kept in a tension-free state, thereby avoiding fracture displacement or loss of immobilization due to high tension. (2) Early functional exercise. The external immobilization device achieves overall stability through 3D-space immobilization. Since there is no tension at the fracture end, the patient can move the knee on the bed immediately after surgery, then ambulate with a walker or crutches the day after surgery, avoiding immobilization-related complications and joint stiffness. (3) Small surgical trauma. No excessive periostea or soft tissues are stripped off during open reduction, and the Kirschner wires used to immobilize the patella are thin and far away from the fracture end, so fracture healing can be facilitated to the greatest extent. (4) Easy removal of the external immobilization device. The external immobilization device can be completely removed in the outpatient clinic after fracture healing. First, the external immobilization ring and the threaded needle are removed, one end of the Kirschner wire is cut off and sterilized, and then the wire is withdrawn through the other end, avoiding re-operation under anesthesia.

Precautions for surgery: (1) When the fracture end is cut and exposed, no excessive periostea and soft tissues can be stripped off. The fracture end is sutured in a tension-free state and in a knee-extension position. If the avulsion fracture blocks are large, the fracture immobilization and reduction can be performed using olive-tipped needles or Kirschner wires with a blocking head. The wire can be fixed with the patellar 1/2 ring using the vertical linker. (2) To immobilize the patellar end, the waist segment of the patella is most often selected because it is the most “hypertrophic” part of the patella. Two Kirschner wires are cross-inserted percutaneously on the cross-section of the waist, in a medial upper-lateral lower and lateral upper-medial lower direction, respectively. The two Kirschner wires should be on the same cross-section and not damage the articular surface, and the skin at the entry point should be free of tension. (3) To immobilize the tibial end, one 2.0-mm Kirschner wire is usually inserted horizontally below the tibial tubercle, and one threaded needle is inserted vertically in front of the tibia on this plane to fix the 1/2 ring. This plane is more conducive to the installation of the joint hinge. (4) When installing the joint hinge, make the knee joint of the patient in the straight position, and install the joint hinge on the lower sides of the patella at the height of the middle axial plane of the patella. During the operation, appropriate adjustments should be made according to the movement of the knee joint to ensure that the movement of the knee joint is consistent with that of the hinge and that the fracture end is in a tension-free state.

In conclusion, tension-free external immobilization is a safe and feasible surgical treatment method for IPFP, and it can benefit the early functional exercise with a satisfactory clinical effect. Even so, there are some deficiencies in this technique. For example, the external immobilization device can bring inconvenience to the patient’s life, especially in winter. Skin pinholes increase the risk of infection. Although the tension-free state can enhance the patellar fracture healing, the tension of the proximal patellar tendon may be increased. The sample of this study was small, and there may be a certain bias in the assessment of clinical efficacy, so larger studies are needed.

## Data Availability

The datasets generated and/or analyzed during the current study are not publicly available because the data are confidential, but the data are available from the corresponding author on reasonable request.

## Abbreviation

IPFP: Inferior pole fracture of the patella
